# The J-curve Association between Systolic Blood Pressure and Clinical Outcomes in Ischemic Stroke or TIA: The BOSS Study

**DOI:** 10.1038/s41598-017-10887-w

**Published:** 2017-10-25

**Authors:** Xuewei Xie, Jie Xu, Hongqiu Gu, Yongli Tao, Pan Chen, Yilong Wang, Yongjun Wang

**Affiliations:** 1 Department of Neurology, Beijing Tiantan Hospital, Capital Medical University, Beijing, China; 2 China National Clinical Research Center for Neurological Diseases, Beijing, China; 3 Center of Stroke, Beijing Institute for Brain Disorders, Beijing, China; 4 Beijing Key Laboratory of Translational Medicine for Cerebrovascular Disease, Beijing, China; 5 Department of Neurology, The First Affiliated Hospital of Zhengzhou University, Henan, China

## Abstract

We aimed to assess the association between systolic blood pressure (SBP) and clinical outcome in 2,397 ischemic stroke (IS) or transient ischemic attack (TIA) patients from the Blood Pressure and Clinical Outcome in TIA or Ischemic Stroke (BOSS) study. BOSS study was a hospital-based, prospective cohort study. The SBP was defined as mean value of 90 days self-measured SBP after onset. Cox proportional hazards models were conducted to test the risk of combined vascular events (CVE) and stroke recurrence among different SBP categories. Restricted cubic splines were used to explore the shape of associations between SBP and clinical outcomes. A J-shaped association of SBP with CVE and stroke recurrence within 90 days was observed (*P* nonlinearity < 0.001 for both). After adjusting for age, gender, medical history, atrial fibrillation, admission NHISS score, and secondary prevention. The hazard ratios (95% confidence intervals) of SBP <115 and ⩾165 mmHg compared with 125–134 mmHg were 3.45 (1.11–10.66) and 7.20 (2.91–17.80) for CVE, 2.68 (0.75–9.53) and 9.69 (3.86–24.35) for stroke recurrence, respectively. Similar J-shaped relationships were found after 1 year of follow-up. In conclusion, both high and low SBP are associated with poor prognosis in this population.

## Introduction

Stroke is one of the most common cause of death and disability in China and worldwide^1,2^. Hypertension has been widely known to be the most common and leading risk factor for stroke^3–5^. However, the relationship between post-stroke blood pressure (BP) and stroke outcomes is a controversial issue.

Compelling clinical trials have documented that BP lowering reduces incidence of mortality from stroke in patients with hypertension^6,7^ or cardiovascular disease in patients without hypertension^8^. In addition, clinical trials have also confirmed that antihypertensive treatment reduces the risk of stroke among individuals with a history of stroke or transient ischemic attack (TIA), including patients with normal BP^9^. BP control has become an important approach for the primary and secondary prevention of stroke among patients with and without hypertension^10^. Despite BP lowering is effective for secondary prevention in patients with cerebrovascular disease, the optimal BP level in this high-risk population remains uncertain. However, we haven’t explored what is the optimal BP range after stroke onset.

Previous studies generated inconsistent results on the association between BP and stroke. For example, some studies described a linear relationship, while others suggested a J-curve or U-curve relationship between BP and stroke outcomes. Whether a lower level of BP is associated with increased risk of poor outcomes is uncertain. Furthermore, few studies described the relationship between self-measured blood pressure (SMBP) and clinical outcomes among ischemic stroke (IS) or TIA patients. Therefore, in the present study with data from the Blood Pressure and Clinical Outcome in TIA or Ischemic Stroke (BOSS) study, we aimed to test the risk of combined vascular events (CVE) and stroke recurrence among different systolic blood pressure (SBP) categories.

## Results

### Baseline characteristics

**Figure 1:**
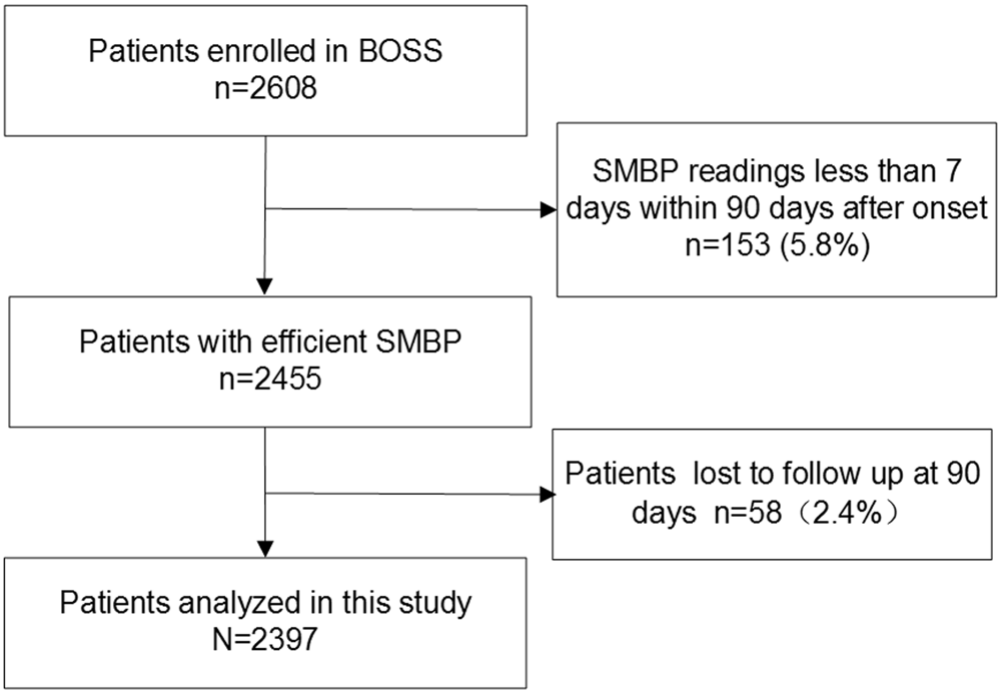
Patient Flow Chart. BOSS indicates Blood Pressure and Clinical Outcome in TIA or Ischemic Stroke; SMBP, self-measured blood pressure.

Among 2,608 patients included in the BOSS study, 153 (5.8%) patients without enough effective SMBP readings within 90 days after onset were excluded, additional 58 (2.4%) lost to follow up at 3 months. Finally, a total of 2397 IS/TIA patients were included in the present analysis (Fig. 1). The baseline characteristics of all patients are listed in Table S1. The patients included and those not included in this analysis were well-balanced except for a lower proportion of history of diabetes mellitus and anti-diabetic therapy. The characteristics of the seven subgroups, defined by SBP levels of <115,115 to 124, 125 to 134, 135 to 144, 145 to 154, 155 to 164 and ≥165 mmHg, are summarized in Table 1. There were 946 (39.5%) patients with age ≥65 and 777 (32.4%) female patients. Patients with higher SBP were older and had higher level of body mass index. In addition, they were more likely to have history of stroke, TIA, hypertension, and diabetes mellitus, and higher proportion of hypertension, diabetes mellitus and coronary heart disease with discharge diagnosis.

**Table 1:** Baseline Characteristics of the Study Population by SBP Category.

	<115 mmHg n = 64	115–124 mmHg n = 248	125–134 mmHg n = 700	135–144 mmHg n = 839	145–154 mmHg n = 419	155–164 mmHg n = 87	≥165 mmHg n = 40	*P* value
Age								<0.0001
<65	54 (84.4)	173 (69.8)	437 (62.4)	489 (58.3)	225 (53.7)	50 (57.5)	23 (57.5)	
≥65	10 (15.6)	75 (30.2)	263 (37.6)	350 (41.7)	194 (46.3)	37 (42.5)	17 (42.5)	
Female	21 (32.8)	74 (29.8)	215 (30.7)	280 (33.4)	142 (33.9)	30 (34.5)	15 (37.5)	0.7975
Current or previous smoker	31 (48.4)	101 (40.7)	306 (43.7)	367 (43.7)	169 (40.3)	37 (42.5)	17 (42.5)	0.8723
Current or previous drinking	24 (37.5)	78 (31.5)	233 (33.3)	262 (31.2)	116 (27.7)	26 (29.9)	9 (22.5)	0.6435
Body mass index, mean ± SD	24.1 ± 2.8	24.5 ± 3.6	24.7 ± 3.2	25.1 ± 3.6	25.1 ± 3.2	24.6 ± 2.9	24.8 ± 3.1	0.0309
History of stroke	18 (28.1)	46 (18.5)	134 (19.1)	192 (22.9)	127 (30.3)	29 (33.3)	14 (35.0)	<0.0001
History of TIA	1 (1.6)	5 (2.0)	30 (4.3)	31 (3.7)	22 (5.3)	2 (2.3)	1 (2.5)	0.0108
History of HTN	33 (51.6)	119 (48.0)	454 (64.9)	639 (76.2)	340 (81.1)	73 (83.9)	31 (77.5)	<0.0001
History of DM	10 (15.6)	37 (14.9)	136 (19.4)	188 (22.4)	101 (24.1)	27 (31.0)	13 (32.5)	0.0035
NIHSS score at admission								0.1466
≤4	51 (79.7)	199 (80.2)	539 (77.0)	634 (75.6)	313 (74.7)	66 (75.9)	27 (67.5)	
5–15	9 (14.1)	47 (19.0)	142 (20.3)	184 (21.9)	97 (23.2)	18 (20.7)	12 (30.0)	
DM with discharge diagnosis	13 (20.3)	55 (22.2)	174 (24.9)	248 (29.6)	127 (30.3)	31 (35.6)	14 (35.0)	0.0202
HTN with discharge diagnosis	41 (64.1)	145 (58.5)	575 (82.1)	786 (93.7)	393 (93.8)	83 (95.4)	39 (97.5)	<0.0001
Dyslipidemia with discharge diagnosis	28 (43.8)	92 (37.1)	306 (43.7)	326 (38.9)	183 (43.7)	33 (37.9)	20 (50.0)	0.1959
CHD with discharge diagnosis	2 (3.1)	23 (9.3)	92 (13.1)	125 (14.9)	44 (10.5)	16 (18.4)	5 (12.5)	0.0142
AF with discharge diagnosis	4 (6.3)	11 (4.4)	33 (4.7)	34 (4.1)	10 (2.4)	2 (2.3)	0 (0.0)	0.3072
Secondary prevention								
antiplatelet	60 (93.8)	225 (90.7)	660 (94.3)	788 (93.9)	394 (94.0)	79 (90.8)	39 (97.5)	0.4276
anti-hypertension	26 (40.6)	102 (41.1)	424 (60.6)	618 (73.7)	331 (79.0)	70 (80.5)	30 (75.0)	<0.0001
lowering-lipid	51 (79.7)	206 (83.1)	607 (86.7)	708 (84.4)	348 (83.1)	69 (79.3)	34 (85.0)	0.4221
Antidiabetic	8 (12.5)	44 (17.7)	130 (18.6)	178 (21.2)	96 (22.9)	24 (27.6)	10 (25.0)	0.1075

DM indicates Diabetes mellitus; HTN, hypertension; CHD, Coronary Heart Disease; AF, Atrial fibrillation.

### SBP and Clinical Outcomes

**Figure 2:**
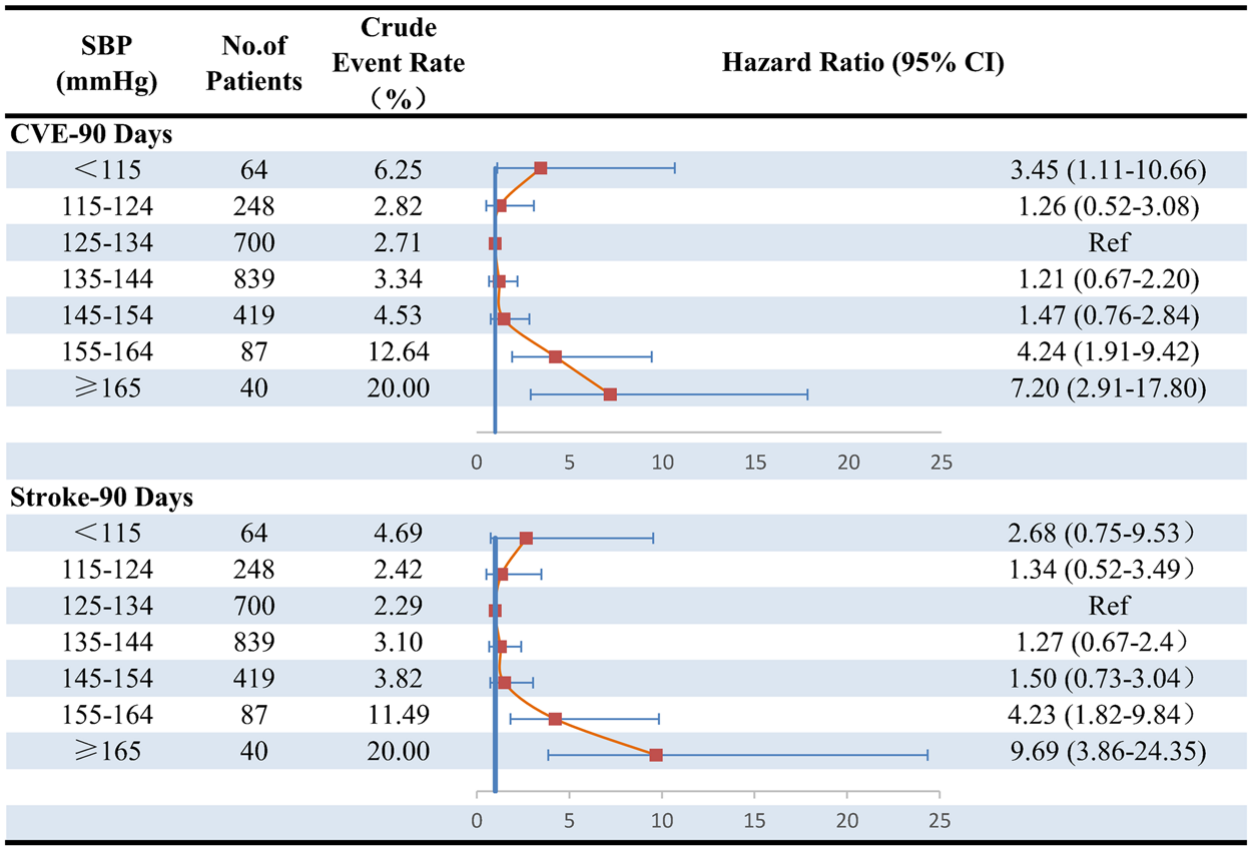
Adjusted Hazard Ratios among SBP Categories for Cumulative Incidence of Outcomes within 90 Days. HR indicates hazard ratio; CI, confidence interval. Adjusted hazard ratios were estimated with adjustment for age, gender, medical history (hypertension, diabetes mellitus, and dyslipidemia), atrial fibrillation, admission NHISS score, and secondary prevention (anti-platelet, anti-lipid, and anti-hypertension).

**Figure 3:**
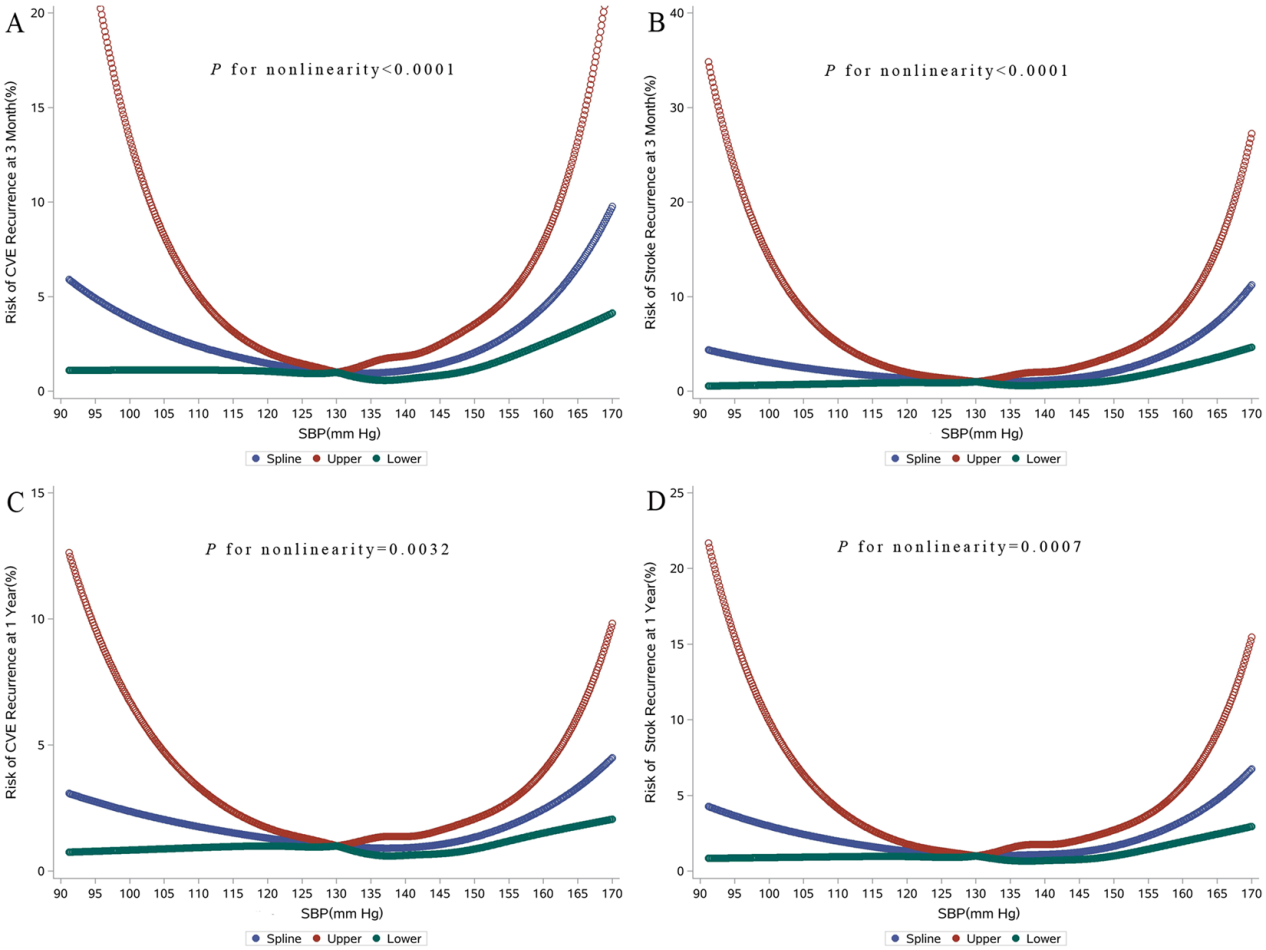
Continuous Hazard Ratios for Clinical Outcomes within 90 Days and 1 Year. Continuous hazard ratios and 95% confidence intervals for CVE (**A**), stroke recurrence (**B**) within 90 days, and CVE (**C**), stroke recurrence (**D**) at 1 year. A test on the relationship between the clinical outcome and SBP levels gave a significant result for nonlinearity (**A**) P < 0.0001, (**B**) P < 0.0001, (**C**) P = 0.0032, **D**: P = 0.0007).

During 3 months of follow-up, a total of 96 (4.0%) patients with CVE and 85 (3.5%) patients with recurrence stroke were identified. The lowest rates of CVE and recurrent stroke were both in SBP 125–134 mmHg group (Fig. 2). After adjusting for age, gender, medical history, admission NHISS score, and other covariates, HRs (95% CIs) of SBP <115 and ≥165 mmHg compared with 125 to 134 mmHg were 3.45 (1.11–10.66) and 7.20 (2.91–17.80) for CVE, 2.68 (0.75–9.53) and 9.69 (3.86–24.35) for stroke recurrence, respectively. Multivariable-adjusted spline regression models showed a J-shaped association of SBP with CVE and stroke recurrence (*P* nonlinearity < 0.001 for both, Fig. 3).

**Figure 4:**
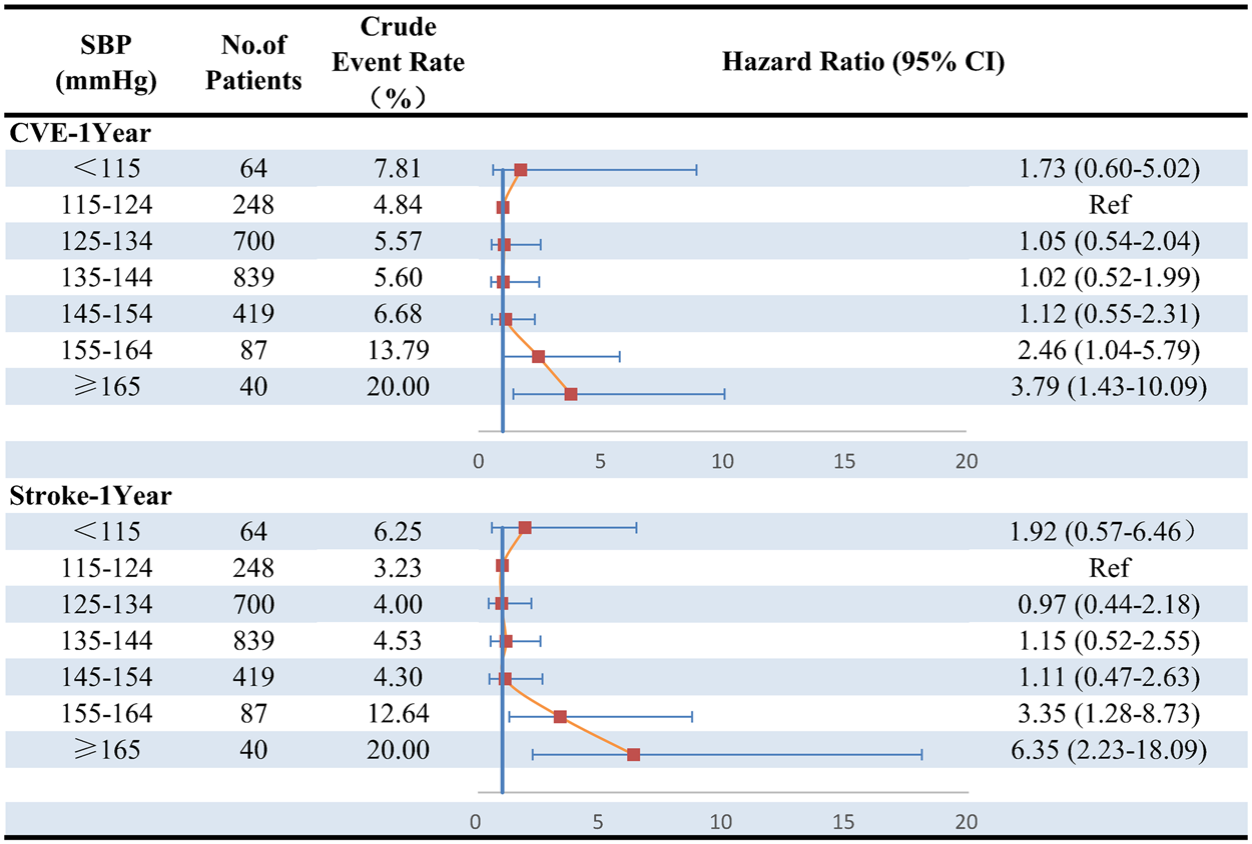
Adjusted Hazard Ratios among SBP Categories for Cumulative Incidence of Outcomes at 1 Year. HR indicates hazard ratio; CI, confidence interval. Adjusted hazard ratios were estimated with adjustment for age, gender, medical history (hypertension, diabetes mellitus, and dyslipidemia), atrial fibrillation, admission NHISS score, and secondary prevention (anti-platelet, anti-lipid, and anti-hypertension).

At 1 year, 151 (6.3%) and 115 (4.8%) patients experienced CVE and stroke recurrence, respectively. The rates of CVE and stroke recurrence were lowest in SBP 115–124 mmHg group (Fig. 4). Multiple-adjusted HR (95% CI) of SBP ≥165 mmHg group was 3.79 (1.43–10.09) for CVE and 6.35 (2.23–18.09) for stroke recurrent, respectively. SBP <115 mmHg did not significantly increased risk of poor clinical outcomes. However, J-shaped relationships between SBP with CVE (*P* nonlinearity = 0.0032) and stroke recurrence (*P* nonlinearity = 0.0007) were observed (Fig. 3).

## Discussion

In this large observational study, we demonstrated a J-curve relationship between SBP and clinical events of CVE and stroke recurrence. Both low and high SBP levels were associated with increased risk of poor clinical outcomes, suggesting that SBP 115–134 mmHg could be the optimal range for IS/TIA patients.

The relationship between SBP and clinical outcomes in the patients with stroke reported in previous studies were conflicting^11–14^. A retrospective study of 368 stroke patients with a history of hypertension found that the recurrence rate had a J-curve relationship to post-stroke diastolic blood pressure but not to post-stroke systolic blood pressure^11^. But another post hoc analysis of International Stroke Trial (IST) found a U-shaped relationship between baseline SBP and clinical outcomes^15^. However, Michelle P. Lin *et al*. found that compared with the normal BP group, the risk of all-cause and vascular mortality tended to be higher in low to normal BP group but was not statistically significant^12^. Furthermore, the analysis of the Prerindopril Protection Against Recurrent Stroke Study (PROGRESS) provided no evidence of an increase in recurrent stroke associated with larger reductions in SBP produced by treatment among patients with cerebrovascular disease^16^. The Systolic Blood Pressure Intervention Trial (SPRINT) suggested that intensive blood pressure management can save lives. But this trial was lack of generalizability to previous stroke patients, who were not included in the study^17^. Despite this controversy showed in above studies, evidence for the existence of J-curve phenomenon in the relationships between SBP and clinical outcomes in IS/TIA population strengthened.

The previous large scale studies were mainly derived from retrospective subanalysis of randomized controlled trials. These studies were not original designed to investigate the effect of the BP on the clinical outcomes but to exam the efficacy and safety of a certain treatment in a prespecified population. Most studies demonstrated that a high post-stroke BP was significantly associated with unfavorable clinical outcomes in patients with acute IS^13^. But the discussion about the relationship between low post-stroke BP and clinical outcomes was still debatable. The explanation for the association between low SBP and poor outcome may be related to the fact that cerebral blood flow is mainly a systolic event. After the acute phase of IS, the autoregulatory mechanism of the cerebral blood flow may be disrupted. Lower BP in this period may lead to the expansion of the ischemic zone surrounding an ischemic, thrombotic or embolic stroke. And poor clinical outcomes may occur. In additional, lower SBP was a marker for potential low state of health. Another interesting finding in our study was that the optimal level of SBP for favorable outcomes is different at two time points. At 3 months, those patients with SBP of 125–134 mmHg had lowest risk of stroke recurrence and CVE. While at 1 year, SBP of 115–124 mmHg would have nadir risk. This inconsistent hinted that as time goes, there are incremental clinical benefits in patients with lower SBP values.

Our study used a novel way to describe post-stroke BP. The SMBP can avoid stress response in initial stage after stroke onset and represent the real BP level. Compared to office and ambulatory BP monitoring, SMBP is convenient to be used by patients themselves at anywhere and anytime. SMBP is already a popular way for self-management in BP after stroke. And the relationship between SMBP and outcomes need to be assessed in more trials.

Our study also has some limitations. First, the results applied to IS/TIA patients should not necessarily be extrapolated to all strokes. Further prospective studies conducted among different populations are needed to replicate our findings. Second, the self-measured BP is less standardized and accurate, but it is more readily applicable to community practice. Third, the possibility of residual confounding cannot be fully eliminated in an observational study, although several important potential confounders have been controlled in multivariable adjusted models. Fourth, we didn’t have data on genetic background of studied population. In future study, important polymorphisms linked to hypertension and stroke should be discussed. Moreover, 211 patients were excluded in this analysis. This may further limit the generalizability of our findings. However, most baseline characteristics were well-balanced between participants includes and excluded.

In summary, the SMBP is practically and widely used in secondary prevention of IS/TIA patients. However, it is rarely reported in clinical trial. The result of this study showed there was a J-curve phenomenon between SBP and risk of CVE and recurrent stroke. Further randomized controlled trials are needed to test in our findings.

## Methods

### Study design and participants

This study was conducted among patients from the BOSS study, a nationwide, hospital-based, longitudinal cohort. The design of the BOSS study has been described in detail elsewhere^18^. Briefly, 2608 patients from 61 hospitals were recruited between October 2012 and February 2014, aimed to assess BP parameters and clinical outcomes in IS/TIA patients. All participants were met the following inclusion criteria, including age of 18 years or older, diagnosed with acute IS or TIA, and within 7 days of the index event. We included participants who had 7 days or more of SMBP readings within 90 days after stroke onset^19^.

The study was approved by the Institutional Review Board at Beijing Tiantan Hospital, as well as ethical committees at the 61 participating hospitals, in compliance with the Declaration of Helsinki. All patients or their legal authorized representatives signed informed consent before participation.

### Blood pressure measurements and classification

After enrollment, a validated monitor (HEM-4030, OMRON Life Science Co. Ltd, Kyoto, Japan) was assigned to each participant. An appropriately sized cuff, containing the correct sized inflatable bladder, was used. Participants themselves or their accompanying relatives were trained to self-monitor blood pressure according to a standard measurement method recommended by the American Heart Association^20^. BP was measured in triple times at each assessment, with an interval of at least 2 min and after 5 min of quiet rest in the seated position. In addition, participants will be advised to avoid alcohol, cigarettes, coffee/tea, and exercise for ≥30 minutes before their BP measurement. The mean of the three measurements was used. Blood pressure measured in the ward during hospitalization and at home after discharge were used for analysis. During hospitalization, BP would be assessed twice a day and BP data were recorded on assigned hospitalization BP diary (first assessment in the morning during 6 AM to 9 AM and second in the evening during 6 PM to 9 PM). At discharge, the assigned OMRON BP monitor was taken home by the participants. They were requested to persistently assess BP twice a day (first assessment in the morning during 6 AM to 9 AM and second in the evening during 6 PM to 9 PM) at home from the first day after discharge to 3 months after onset. BP readings were recorded on the assigned home BP diaries. SMBP was defined as regular measurement of blood pressure by the patients themselves or their accompanying relatives both in and out of clinical setting. SMBP should be monitored for 7 days or more, with at least two morning and two evening measurements^21^. The average of at least 14 values, taken in the morning and the evening, was used. The mean value of SMBP within 90 days after onset and before clinical events was used for data analysis. The SBP values were categorized by 10 mmHg increments: <115, 115 to 124, 125 to 134, 135 to 144, 145 to 154, 155 to 164, and ≥165 mmHg.

### Clinical outcomes assessment

The patients were followed up in person at 3 months and by telephone at 12 months. We would call back patients with nonfatal events for a face-to-face follow-up or carry out a home visit. The clinical outcomes were CVE and recurrent stroke (ischemic or hemorrhagic) at 3 months and 1 year after onset. CVE were composed of IS, hemorrhagic stroke, myocardial infarction, or vascular death. Vascular death included fatal stroke, fatal myocardial infarction, and other cardiovascular death. Death certificates were obtained for deceased participants, and hospital data were abstracted for all vascular events. Recurrent stroke was defined as a new neurological deficit or a deterioration of the previous deficit that fits the definitions for ischemic or hemorrhagic stroke^22^.

### Statistical analysis

The categorical characteristics of patients were reported as frequency (percent) and were compared using chi-square test, while continuous variables are expressed as SD and were compared using analysis of variance. Cox proportional hazards models were used to estimate the effects of SBP on clinical events. The crude and multiple-adjusted hazard ratios (HRs) and corresponding 95% confidence intervals (CIs) were assessed for each category of SBP using SBP of 125 to 134 mmHg or 115 to 124 mmHg as the reference group. The covariates included in the multivariable model were age, gender, medical history (hypertension, diabetes mellitus, and dyslipidemia), atrial fibrillation, admission NHISS score, and secondary prevention (anti-platelet, anti-lipid, and anti-hypertension). In addition, we used restricted cubic splines to explore the shape of the associations between SBP and clinical outcomes. Data were analyzed using SAS version 9.4 (SAS Institute Inc, Cary, NC). All P values were 2-tailed, and a significance level of 0.05 was used.

## Electronic supplementary material

Supplemental file
