# Supplementary material for: The J-curve Association between Systolic Blood Pressure and Clinical Outcomes in Ischemic Stroke or TIA: The BOSS Study
Source: Sci Rep. 2017 Oct 25;7:14023. doi: 10.1038/s41598-017-10887-w (PMC5656684; doi:10.1038/s41598-017-10887-w)
Supplement: Supplementary file 1 — Supplemental file [file 41598_2017_10887_MOESM1_ESM.pdf]

TITLE PAGE

TITLE:

The J-curve Association between Systolic Blood Pressure and Clinical Outcomes in Ischemic Stroke or TIA: The BOSS Study

SHORT TITLE: Systolic Blood Pressure and Clinical Outcomes

AUTHOR LIST

Xuewei Xie, Jie Xu, Hongqiu Gu, Yongli Tao, Pan Chen, Yilong Wang, Yongjun Wang

Table S1. Baseline characteristics of patients included vs. excluded in the study

	**Patients Included n=2,337**	**All other Patients n=211**	***P* value**
Age			0.3643
<65	1451 (60.5)	121 (57.3)	
≥65	946 (39.5)	90 (42.7)	
Female	777 (32.4)	68 (32.2)	0.9554
Current or previous smoker	1028 (42.9)	96 (45.5)	0.2646
Current or previous drinking	748 (31.2)	70 (33.2)	0.5236
Body mass index，mean ± SD	24.9±3.4	24.6±3.6	0.3468
History of stroke	560 (23.4)	58 (27.5)	0.0479
History of TIA	92 (3.8)	10 (4.7)	0.1413
History of HTN	1689 (70.5)	148 (70.1)	0.9629
History of DM	512 (21.4)	56 (26.5)	0.1129
NIHSS score at admission			0.0438
≤4	1829 (76.3)	138 (65.4)	
5-15	509 (21.2)	56 (26.5)	
DM with discharge diagnosis	662 (27.6)	77 (36.5)	0.0057
HTN with discharge diagnosis	2062 (86.0)	176 (83.4)	0.3298
Dyslipidemia with discharge diagnosis	988 (41.2)	95 (45.0)	0.2718
CHD with discharge diagnosis	307 (12.8)	21 (10.0)	0.2338
AF with discharge diagnosis	94 (3.9)	10 (4.7)	0.5559
Secondary prevention			
antiplatelet	2245 (93.7)	189 (89.6)	0.3820
anti-hypertension	1601 (66.8)	130 (61.6)	0.6481
lowering-lipid	2023 (84.4)	173 (82.0)	0.3219
Antidiabetic	490 (20.4)	57 (27.0)	0.0064

DM indicates Diabetes mellitus; HTN, hypertension; CHD, Coronary Heart Disease; AF, Atrial fibrillation.
